# Theoretical assessment of feasibility to sequence DNA through interlayer electronic tunneling transport at aligned nanopores in bilayer graphene

**DOI:** 10.1038/srep17560

**Published:** 2015-12-04

**Authors:** Jariyanee Prasongkit, Gustavo T. Feliciano, Alexandre R. Rocha, Yuhui He, Tanakorn Osotchan, Rajeev Ahuja, Ralph H. Scheicher

**Affiliations:** 1 Division of Physics, Faculty of Science, Nakhon Phanom University, Nakhon Phanom 48000, Thailand; 2 Nanotec-KKU Center of Excellence on Advanced Nanomaterials for Energy Production and Storage, Khon Kaen 40002, Thailand; 3 Institute of Chemistry, Physical Chemistry Department, Universidade Estadual Paulista (UNESP), Araraquara, SP, Brazil; 4 Instituto de Física Téorica, Universidade Estadual Paulista (UNESP), São Paulo, SP, Brazil; 5 School of Optical and Electronic Information, Huazhong University of Science and Technology, LuoYu Road, Wuhan 430074, China; 6 Department of Physics, Faculty of Science, Mahidol University, Bangkok, 10400, Thailand; 7 Applied Materials Physics, Department of Materials and Engineering, Royal Institute of Technology, SE-100 44 Stockholm, Sweden; 8 Division of Materials Theory, Department of Physics and Astronomy, Uppsala University, Box 516, SE-751 20 Uppsala, Sweden

## Abstract

Fast, cost effective, single-shot DNA sequencing could be the prelude of a new era in genetics. As DNA encodes the information for the production of proteins in all known living beings on Earth, determining the nucleobase sequences is the first and necessary step in that direction. Graphene-based nanopore devices hold great promise for next-generation DNA sequencing. In this work, we develop a novel approach for sequencing DNA using bilayer graphene to read the interlayer conductance through the layers in the presence of target nucleobases. Classical molecular dynamics simulations of DNA translocation through the pore were performed to trace the nucleobase trajectories and evaluate the interaction between the nucleobases and the nanopore. This interaction stabilizes the bases in different orientations, resulting in smaller fluctuations of the nucleobases inside the pore. We assessed the performance of a bilayer graphene nanopore setup for the purpose of DNA sequencing by employing density functional theory and non-equilibrium Green’s function method to investigate the interlayer conductance of nucleobases coupling simultaneously to the top and bottom graphene layers. The obtained conductance is significantly affected by the presence of DNA in the bilayer graphene nanopore, allowing us to analyze DNA sequences.

Sequencing DNA is the first step on the route towards so-called personalized or precision medicine^1^,^2^ as well as fields of research where genetic information is the key. Although there have been advances in low-cost fast sequencing to the point of claims for $1000-per-genome sequencing^3^,^4^, this has been largely due to scaling of existing processes. In search of new possibilities, the use of nanopore technologies has gained momentum^5^. The general idea is to drive the DNA molecule through a tiny hole in a biological^6^,^7^,^8^ or solid-state^9^,^10^,^11^ membrane while measuring the ionic current in the solution^12^,^13^ or the transverse current^14^,^15^ being conducted in the membrane itself. The first approach utilizes the fact that the magnitudes of ionic current blockage vary significantly when different types of nucleotides pass through the biological nanopore^6^,^7^,^8^,^12^,^13^. Such variations are caused by the different binding strengths between nucleotides and biological pore wall, and can serve as the electronic signatures of target nucleobases. On the other hand the second strategy, namely the transverse conductance approach, is based on the principle that the difference between electronic structures of the four types of nucleobases can be discriminated by measuring the conductance through them^14^. Experimentally, it was demonstrated that by using mechanically controllable break junctions, cytosine, thymine and guanine could be statistically identified according to the different amplitudes of tunneling currents^16^. The results were interpreted through the highest occupied molecular orbitals (HOMO) or lowest unoccupied molecular orbitals (LUMO) of the nucleobases. The method was further developed towards multidetection of molecules with different electronic structures^17^, such as partial sequencing of peptides^18^.

In either approach, graphene has been heralded as an ideal candidate for a one-atom-thin membrane^19^,^20^,^21^ for DNA sequencing, first in the form of nanogaps^22^, and subsequently using pores^21^,^23^,^24^,^25^,^26^,^27^. Graphene nanogaps for DNA sequencing^22^ have been proposed as promising candidates for next-generation DNA sequencing, but some difficulties in their fabrication and application still remain. From previous studies^28^,^29^, very low tunneling current across the gap has been found, resulting from, firstly, the highest occupied molecular orbital (HOMO) level of nucleobases being located far away from the Fermi level of graphene, and secondly, the nucleobases being weakly coupled to the graphene electrodes.

One possibility to simplify the fabrication process and simultaneously increase the transverse current is the use of a graphene nanopore to identify single nucleobases^19^,^30^,^31^,^32^; DNA translocating through the graphene nanopore can modulate the edge current in graphene nanoribbons (GNRs)^25^,^33^. However, the nanopore diameter could play an important role in electronic transport; the sensitivity of electrical nucleobase detection significantly decreases as the nanopore size is increased from 1.2 nm to 1.7 nm^34^.

In this work, we address these issues by investigating an alternative setup, namely a pair of aligned nanopores in bilayer graphene arranged in the Bernal (AB) stacking. The upper layer and bottom layer of graphene act as two separate electrodes which are connected by nucleobases traveling through the nanopores (see Fig. 1). The pore edges were passivated by OH-groups which might be a realistic scenario for wet-lab experiments^35^,^36^. In this way, He *et al.*^37^ recently explored the feasibility of a graphene/hexagonal boron nitride (h-BN)/graphene nanopore setup for single molecule detection. Using a precision transfer technique, Chae *et al.*^38^ have fabricated separate contacts on each layer of two stacked graphene monolayers and measured its interlayer conductance. Previously, Sadeghi *et al.*^39^ have investigated the potenital of bilayer-graphene nanopores for DNA sequencing applications; however, using the interlayer conductance between the two graphene layers for sequencing purposes has not been considered yet. As compared to monolayer graphene, the density of states of bilayer graphene is higher, giving rise to more conductance channels in transport^40^. Furthermore, by increasing the number of stacked graphene layers, it might be possible to prolong the translocation time of DNA^41^,^42^.

As the DNA passes through the pore, there are many possibilities of molecular orientations with respect to the nanopore. We simulated DNA sequencing in a prototypical graphene device using classical molecular dynamics simulations of DNA translocation through the pore under the effect of the environment. In this manner, we are able to analyse the nucleobase trajectories and the interaction between the nucleobases and the nanopore. We further investigated the electrical transport properties of the four nucleobases: guanine (G), adenine (A), cytosine (C), and thymine (T), using a bilayer graphene nanopore for the purpose of electrical contact and nucleobase identification. This was achieved using a combination of classical molecular dynamics simulations, state-of-the-art density functional theory (DFT), and non-equilibrium Green’s functions (NEGF). As the nucleobase from a single-stranded DNA molecule is placed in the bilayer graphene nanopore, the local interlayer conductance is modulated by the nucleotide bridging the two graphene layers, revealing the DNA sequence.

## Results and Discussion

From the classical molecular dynamics (MD) trajectories one can analyse the interaction of the nucleobases with the nanopore. In Fig. 2 we present a histogram of the minimum distance between the nucleobase and carbon atoms on the edges of the nanopore. We notice two trends; A and G - the purines - interact strongly with the edge of the pore and throughout the simulation remain at an average distance of 2 Å. T, on the other hand interacts only weakly with the pore edge, and spends most of its time further away from the edges. C has an intermediate trend. It tends to hydrogen bond to the OH groups on the pore, but not as strongly as A and G, thus for periods during the dynamics simulation, it moves away from the pore edge, but returns after some steps.

We also analyzed the angle *θ*_1_ formed between the normal to the aromatic rings in the nucleobase and the normal to the plane of graphene, and the angle between the projection of the normal to the aromatic rings on the plane of graphene and the vector lying on that same plane, which is normal to the circumference of the nanopore, *θ*_2_. Figure 3 shows that, in line with the results for the distance, C and G have the smallest angular dispersion. This means that the interaction with the edge is relatively strong. Nevertheless, while G lies almost vertically, the angle between the aromatic ring in A and the plane of graphene is approximately 45°. In C, when the base is closer to the edge it is also almost perpendicular to the plane of the bilayer. In all cases, the nucleobases are inclined inside the pore, an indication that they tend to act as a bridge between the two layers of graphene. As we will show below, the binding of the molecule to the edge of the pore can be correlated to transport properties of the system.

The hydrogen bonds are taken into account implicitly by the electrostatic and van der Waals terms of the particular classical force field being used. For the graphene pore edge, parameters for the phenol were taken, in order to determine the charge of the oxygen and hydrogen atoms, as well as the carbon adjacent to the oxygens. The absolute value of the classical partial charge is significantly higher in oxygen, compared to hydrogen, and so, the edge will be a strong hydrogen bond acceptor. This means that adenine and guanine should bind to the edge more strongly, because not only there are more hydrogen donor groups, but also they exhibit the correct spatial arrangement to keep the contact with the two graphene sheets at the same time. Although there are other hydrogen bond donor and acceptor groups available in each nucleobase, not all of them make hydrogen bonds with the pore, at the same time. For the purpose of counting the number of hydrogen bond donors, each hydrogen atom bound to a nitrogen/oxygen atom in a nucleobase is regarded a donor, so the amine groups of the nucleobases donate two hydrogen bonds. The orientation of the A and G nucleobases are closely transversal, with respect to the graphene plane, for G and A bases, because there are two effective hydrogen bond donors in each base, relatively spaced in the molecule, spanning almost all the length of the nucleobase plane, providing an efficient locking of the base position, across the two pores. Particularly for A, there is a potential effective hydrogen bond acceptor, which is the unprotonated nitrogen atom of the base, close to the amine group, which only makes effective contact with the pore if the base is tilted, with respect to the graphene plane. In the C nucleobase, there are also two effective hydrogen bond donors, but located in the same position, in a nitrogen atom, attached to the end of the nucleobase ring. In the T nucleobase, there is only one hydrogen bond donor. Therefore, in both cases the coupling with the graphene pore between the two pores happen only by that atom, and is significantly weaker, causing the disruption of the pore-nucleobase interaction along the MD simulation.

Using the MD simulations, we can trace the nucleobase trajectory as the DNA is translocated through the bilayer graphene nanopore. From simulations for isolated single strands of DNA being pulled, we found that the distance between nucleobases is of the order of 5–7 Å, and thus larger than those observed in a relaxed structure or in double-stranded DNA. At these distances we do not expect significant interference in the electronic transport from bases above or below the base inside the pore. In that sense the system can be regarded to posses single-nucleobase resolution. Snapshots which provide the possibility of strong coupling strength between the nucleobase and nanopore were selected (see Supplementary Information for further details). The quantum transport of such selected configurations was calculated using NEGF technique based on DFT^43^ as implemented in the SMEAGOL package^44^. As the DNA passes through the pore, the interlayer conductance is modulated by a nucleotide bridging the two graphene layers. Note that while the sugar-phosphate backbone, counterions and water molecules were naturally taken into account in the MD simulations, they were excluded from the quantum transport calculations since these entitites are alike for all nucleobases and thus cannot add any distinctive features to the electric signal^33^.

Figure 4 shows the transmission coefficients yielding the maximum conductance of four target nucleobases (G, A, C, T) for selected snapshots, as compared to that in the absence of nucleobases. Note that we compare the conductance among the selected snapshots from MD simulation, providing the shortest distance between the nucleobase and the pore. The effect of distance from the pore on the quantum conductance has been tested; the conductance decreases significantly with increasing distance from the edge resulting from the weakening of nucleobase-graphene coupling (see the Supplementary Information section). Obviously, the presence of nucleobases within the nanopore enhances the transmission through graphene layers. The effect arises from electronic coupling between the nucleobases and OH-pore edge states of bilayer graphene nanopore. Since the empty pore has a much lower transmittance around the Fermi level compared to that in the presence of bases, we expect that the backgroud of leakage current between the graphene layers will not significantly affect the signature nucleobase analysis at low bias voltage. The conductance is seen to be ordered in the following hierarchy: C > G ~ T > A. Interestingly, we see a similar trend as in a previous study^30^ on the conductance of nucleobases translocated through a nanopore in a H-passivated GNR.

The scattering wavefunction of the transmission channel at the Fermi energy is employed to help understand the vertical transport mechanism of such a nanodevice setup. It indicates the pathway taken by the electrons traversing the layered structure. We take the cases of C and G as examples to analyze the scattering state. The real-valued scattering wavefunction indicates an exponential decay into the junction, while the imaginary part of the wavefunction is too small to be observed for most cases^45^. As presented in Fig. 4, the scattering state of the nucleobase, i.e. C and G, coupled to the pore edge of bilayer graphene, indicates the propagating wave function from the top to the bottom layer of bilayer graphene. For T and A, the wave function is too small to be visible in the figure. It is seen that the real part of the scattering state of G rapidly decays compared to that of C. In other words, the incoming wave from the bottom layer of graphene is propagating to the upper layer of graphene, which is mostly reflected in the case of G. We further analyze the transmission spectra of Fig. 4: the resonance peaks associated with the HOMO of isolated nucleobases are identified, as presented in the Supplementary Information. We observed that the quantum transport across the graphene layer is dominated by the HOMO for purines, while that of the pyrimidines is dominated by the LUMO. The high conductance of the pyrimidines is obtained due to the delocalized nature of the LUMO. When a nucleobase travels through the nanopores, the HOMO-LUMO levels are shifted due to the geometric fluctuations of the nucleobase. The conductance is, however, not only determined by the molecular level position, but also by the nucleobases coupling to the graphene layer.

Figure 5 demonstrates the correlation between coupling of the base and transport by presenting the conductance as a function of binding energy showing a means to differentiate between the four nucleobase types. As the DNA is translocated through the bilayer graphene nanopore, the change in transmission profile (shape and peak height) of each nucleobase depends on their positions and orientations within the pore. As a result, the modulation of conductance through alteration of electronic coupling around the bilayer nanopore edge is observed. The rectangles indicate the range of conductance and binding energy values of the four nuclobases as determined from our calculations. For T, a dashed line is used to outline the range because this nucleobase does not bind with the OH-functionalized edges of the bilayer graphene nanopore. Instead, poly(dT)_6_ disconnects from the pore edges in our simulatons after about 5 ns. Thus, only a few snapshots could be selected for transport calculations. For C, there is some conductance overlap with G, but no overlap with A. It is important to emphasize that the conductance interval shown in Fig. 5 should be considered as the maximum conductance variation.

Two different distribution ranges of the conductance for C are observed in Fig. 5. The change of binding sites of C after 4 ns (see the Supplementary Information) leads to changing from the highest to the lowest conductance. Furthermore, T shows a large variation in conductance. According to our MD simulation results, A and G stick to the edge whereas C and T tend to move away from the edge. Because of the weaker interaction in the latter case, there are considerable fluctuations in not only the bridging sites but also the molecular conformations, and subsequently very large fluctuation in conductance for pyrimidine bases.

In summary, we have proposed a fast DNA sequencing device based on reading the interlayer conductance of target nucleobases between the top and bottom graphene layers in a bilayer arrangement. We show that this setup gives rise to significant changes in the conductance for each one of the bases compared to the isolated pore and allows for the differentiation of the bases. We correlated the binding of the bases to the edge of the graphene nanopore saturated with OH groups to the conductance of each base. We also show that the interaction of the purines with the edge of the nanopore leads to a small dispersion of distances from the base to the pore, and to a well-determined orientation. This means that these two bases, for the size of pore studied here, are almost frozen in place. As a consequence the dynamical conductance fluctuations are expected to be relatively small. As for the pyrimidines, when the base is close to the edge, a similar trend is observed, but we also note that, given their size, it is much easier for them to move away from the edge, giving rise to double peaks in the position histograms. The conductance fluctuations of pyrimidines are found to be much larger than that of purines, resulting from the interaction strength between the nucleobase and pore edge. Based on our results, it can be rather difficult to identify C and T for this setup. Functionalizing graphene edges with different functional groups can possibly improve the interaction strength between the pyrimidines and the pore in order to suppress orientational fluctuations^29^,^46^.

## Methods

### MD simulations

A classical molecular dynamics simulation was employed in order to obtain trajectories for the subsequent transport calculations. For this purpose, we set up the system as a DNA homopolymer crossing a pore of a bilayer graphene sheet, surrounded by water and counterions. For the DNA, 4 periodic single strand homopolymers were constructed, each of which containing 6 repetitions of the same base type, namely: poly(dA)_6_, poly(dC)_6_, poly(dG)_6_ and poly(dT)_6_. The bilayer graphene sheet was built in the AB Bernal stacking configuration. The edge of the 2-nm diameter nanopore was passivated by OH-groups.

**Figure 1:**
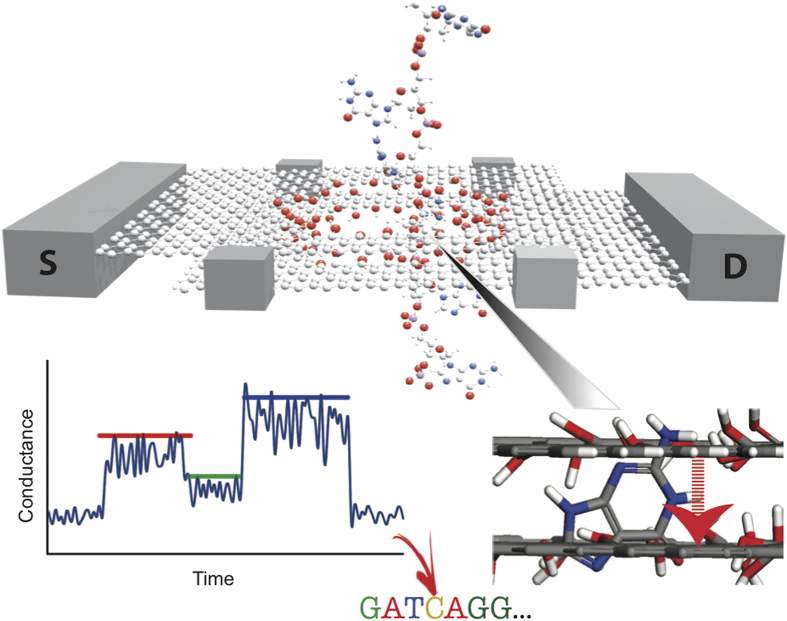
Schematic of a bilayer graphene nanopore-based device for measuring the conductance of the four target nucleobases (G, A, C, T). The bilayer graphene is arranged in the Bernal (AB) stacking configuration. The top and bottom layers of bilayer graphene are used as source and drain electrodes, respectively. The edges of both nanopores were passivated by OH-groups. As DNA is translocating through the pore, the local interlayer conductance is modulated as a function of time by the nucleotide bridging the two graphene layers, as illustrated in the schematic measurement sketch (bottom left panel). The average maximum signal heights (vertical position of the colored bars) along with the signal duration (horizontal length of the colored bars) can then ideally be used to distinguish and identify the nucleotides in DNA.

In order to prevent DNA sticking to the graphene, and to simulate the effect of the coating (usually used in this type of experiment), another layer of non-sticking material was used to cover the two sides of the graphene. A dummy atom was used for this purpose, with van der Waals parameters designed to mimic the presence of a protective coating layer (*σ* = 3.50000e-01 and *ε* = 6.00000e-03). In this 4-layer system, a pore of diameter 2 nm was drilled in the center, and the graphene carbon atoms in the pore were saturated using hydroxyl functional groups. The graphene is periodic in x-direction (see Fig. 1 of the manuscript for details), while the ends of each of the layers in z direction are finite, being saturated with hydrogen atoms.

The homopolymer-graphene system was then placed in a 4 nm × 5 nm × 4 nm periodic cell, and filled with water molecules and counterions. The counterions were placed so the entire system is kept neutral. At the same time, a fixed concentration of 0.1 M was used, so both Na^+^ and Cl^−^ counterions were used. This configuration comprised a total of around 10350 atoms, for each homopolymer, resulting in 4 MD runs. We employed the AMBER99SB force field^47^,^48^ in GROMACS package^49^,^50^,^51^, for the description of the DNA molecule and the counter ions. The SPC model was employed for water description, and for graphene, van der Waals parameters were taken from aromatic fragments in AMBER99SB, and the charges are zero everywhere except at the pore, where the charges are assigned using the same fragments in AMBER99SB in the case of hydroxyl ending, the charges for O-H and the C neighbour atom are taken from phenol and in the hydrogen ending, from benzene.

**Figure 2:**
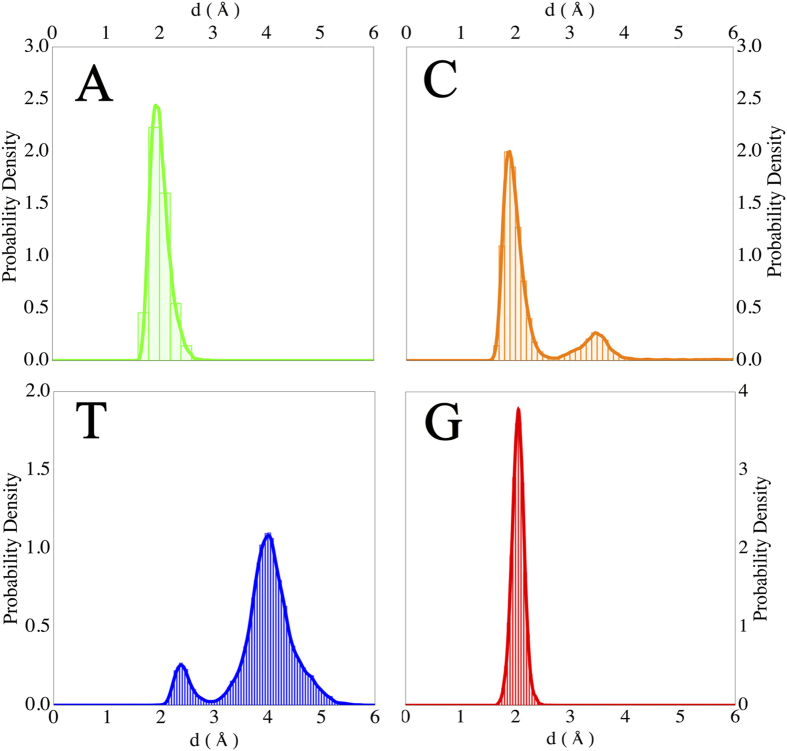
Distribution of the closest distance between atoms in the nucleobase and the edge of the nanopore in bilayer graphene.

**Figure 3:**
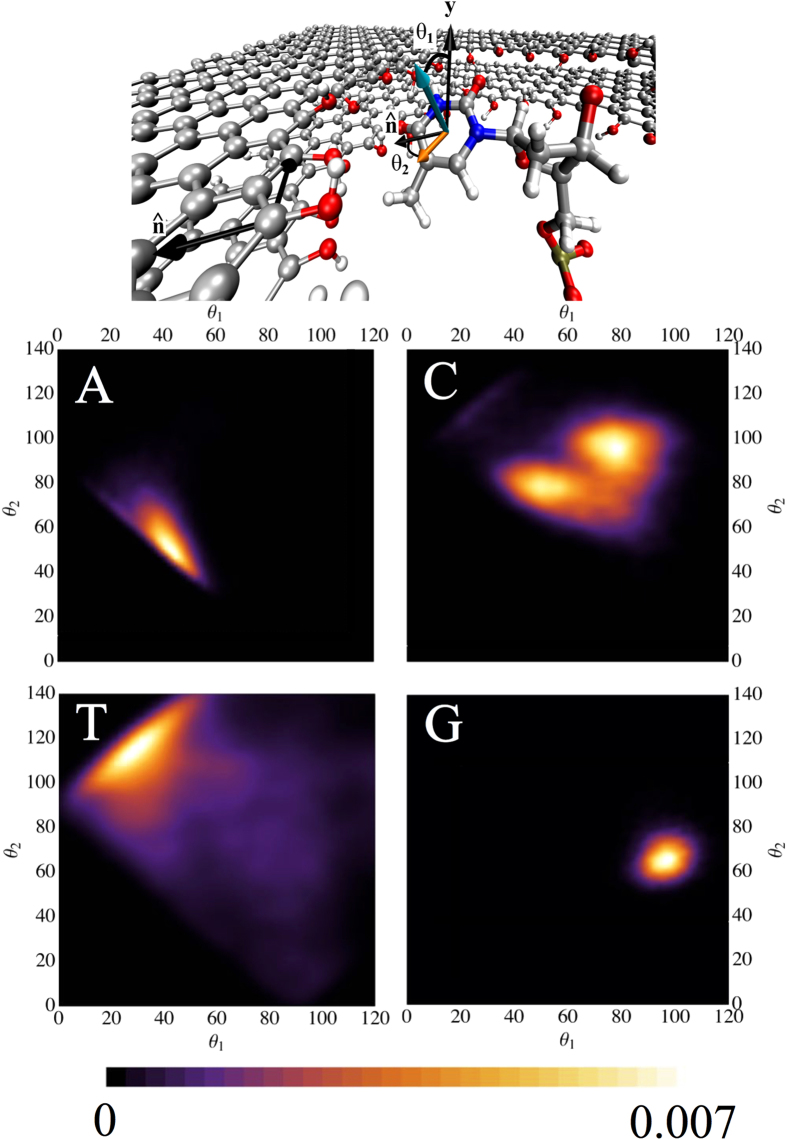
Top panel: Representation of a single snapshot of the dynamics for T. The angle *θ*_1_ is defined as the angle between the normal to the plane of the nucleobase (cyan arrow) and the y axis (the normal to the plane of graphene), and *θ*_2_ is the angle formed between the projection of the normal to the nucleobase onto the plane of graphene (orange arrow), and the vector normal to the circumference of the nanopore, 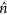. Lower Panels: Histogram of the probability density of angles between each nucleobase and the nanopore in bilayer graphene.

**Figure 4:**
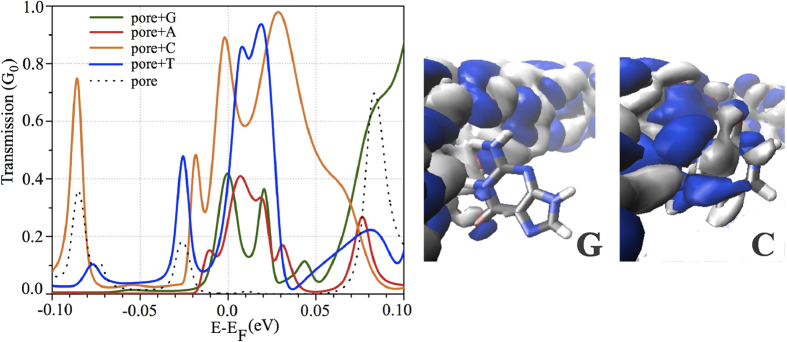
Left panel: The transmission coefficients of the four target nucleobases (G, A, C, T) yielding maximum conductance, as compared to that in the absence of nucleobases. Right panel: The scattering state of the highest conducting channel of C and G shown in the left panel. The colors indicate the two different signs of the real-valued wavefunction.

**Figure 5:**
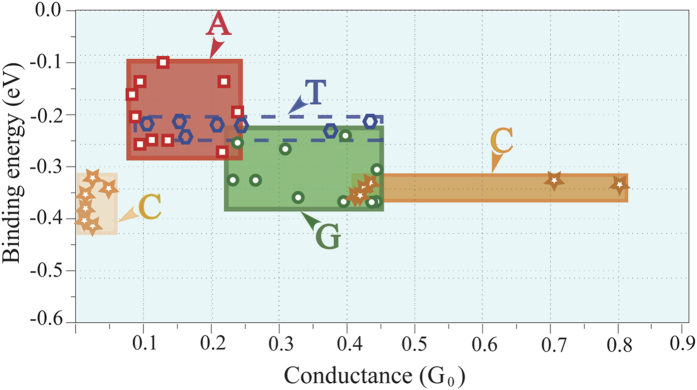
The conductance vs. binding energy of the four target nucleobases (G, A, C, T). Note that we selected some configurations providing strong coupling between the nucleobase and pore edges from MD snapshots. The rectangle represents the region of the conductance vs. binding energy variations of each nucleobase.

A standard MD protocol was applied to this system: 1 ns of thermalization in the NVT ensemble at 300 K, followed by 1 ns of density equilibration in the NPT ensemble at 300 K and 1 atm, and 10 ns for the production run in the NPT ensemble, 300 K/1 atm. In order to obtain Figs 2 and 3 of the main manuscript we continued the production run for an extra 40 ns. The Nosé-Hoover thermostat and Besendsen barostat algorithms were employed in this setup. Note that during data collection period of 10 ns, one snapshot was saved every 2 ps.

The rate of the translocation has not been investigated since the actual translocation event would be of the order of microseconds per base beyond what is attainable by current molecular mechanics methods. We thus considered that the process is quasi-static, i.e., we fixed the height of the backbone of the DNA, but allowed the nucleobase to freely move around the pore during the simulation. We then moved the entire strand once up by 2 Å and once down by 2 Å, and repeated the whole process of MD simulations and quantum transport calculations.

### Quantum transport calculations

We investigated the quantum transport by employing a robust combination of nonequilibrium Green’s function (NEGF) techniques based on DFT^43^ as implemented in the SMEAGOL package^44^ within the generalized gradient approximation (GGA) to the exchange-correlation functional^52^. Each graphene layer is periodic in x-direction, while its ends in z-direction are semi-finite, being saturated by H atoms at the edge. See Supporting Information for additional details on bilayer-graphene nanopore setup for measuring the conductance. The valence electrons are described using a local basis set with a single-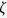 plus polarization orbital (SZP) for all elements, which has already been proven a valid approach for the nucleobase-graphene system^28^,^29^. The atomic core electrons are modeled using Troullier-Martins soft norm-conserving pseudopotentials^53^. We considered only the 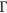-point for Brillouin zone sampling and the mesh cutoff energy is 150 Ry. Within the NEGF approach, we calculate the transmission coefficient, 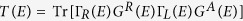, where 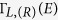 is the broadening matrix of the left (right) electrode, and 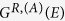 is the retarded (advanced) Green’s function. Then, we simply evaluate the conductance, 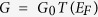, where 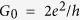 is the quantum conductance, 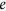 is the charge of the electron and 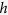 is Planck’s constant.

The DFT simulation was performed with SIESTA^43^ to investigate the adsorption mechanism between the pore and the nucleobase. The exchange-correlation functional and basis sets employed in the DFT calculation are identical to those described above for the transport calculation part. For the k-point mesh generation, 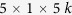-points were employed. The binding energy between the pore and the base was calculated as 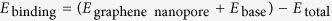, where 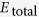 is the total energy of the system (graphene nanopore+base), and 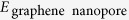 and 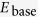 is the energy of graphene nanopore and nucleobase, respectively.

## Additional Information

**How to cite this article**: Prasongkit, J. *et al.* Theoretical assessment of feasibility to sequence DNA through interlayer electronic tunneling transport at aligned nanopores in bilayer graphene. *Sci. Rep.* **5**, 17560; doi: 10.1038/srep17560 (2015).

## Supplementary Material

Supplementary Information
