# Supplementary material for: Theoretical assessment of feasibility to sequence DNA through interlayer electronic tunneling transport at aligned nanopores in bilayer graphene
Source: Sci Rep. 2015 Dec 4;5:17560. doi: 10.1038/srep17560 (PMC4669446; doi:10.1038/srep17560)
Supplement: Supplementary Information [file srep17560-s1.pdf]

Supplementary Information for: Theoretical assessment of feasibility to sequence DNA through interlayer electronic tunneling transport at aligned nanopores in bilayer graphene

Jariyanee Prasongkit [1,2*],Gustavo T. Feliciano [3],Alexandre R. Rocha [4*],Yuhui He [5],Tanakorn Osotchan [6],Rajeev Ahuja [7,8],Ralph H. Scheicher [8*]

[1]*Division of Physics, Faculty of Science, Nakhon Phanom University, Nakhon Phanom 48000, Thailand*

[2]*Nanotec-KKU Center of Excellence on Advanced Nanomaterials for Energy Production and Storage, Khon Kaen 40002, Thailand*

[3]*Institute of Chemistry, Physical Chemistry Department, Universidade Estadual Paulista (UNESP), Araraquara, SP, Brazil*

[4]*Instituto de Física Teórica, Universidade Estadual Paulista (UNESP), São Paulo, SP, Brazil*

[5]*School of Optical and Electronic Information, Huazhong University of Science and Technology, LuoYu Road, Wuhan 430074, China*

[6]*Department of Physics, Faculty of Science, Mahidol University, Bangkok, 10400, Thailand*

[7]*Applied Materials Physics, Department of Materials and Engineering, Royal Institute of Technology, SE-100 44 Stockholm, Sweden*

[8]*Division of Materials Theory, Department of Physics and Astronomy, Uppsala University, Box 516, SE-751 20 Uppsala, Sweden*

S1. Snapshots selection from MD simulations

We selected a reference atom to measure the distance between the reference one and all other atoms of bilayer graphene (see Figure S1). The shortest distances of each snapshot for four nucleobases (guanine, adenine, cytosine, and thymine) were saved, as presented in Figure S2. Some snapshots, providing the strong coupling strength, were selected for subsequent transport calculations as marked with black circles.

A change in the binding site was observed for cytosine (see Figure S2). After 1.5 ns, the cytosine gradually shifts to middle of pore and then moves close to the pore edge at a new binding site. The transport calculation in which the cytosine lies far from the pore edge has been performed, as shown in Figure S4. The transmission function exhibits the shift of peak position relative to the Fermi energy, and the transmission drops due to weakening of nucleobase-graphene coupling. Additionally, the disconnection of thymine to the pore edge was observed after 5 ns and not likely to return close to the edge.

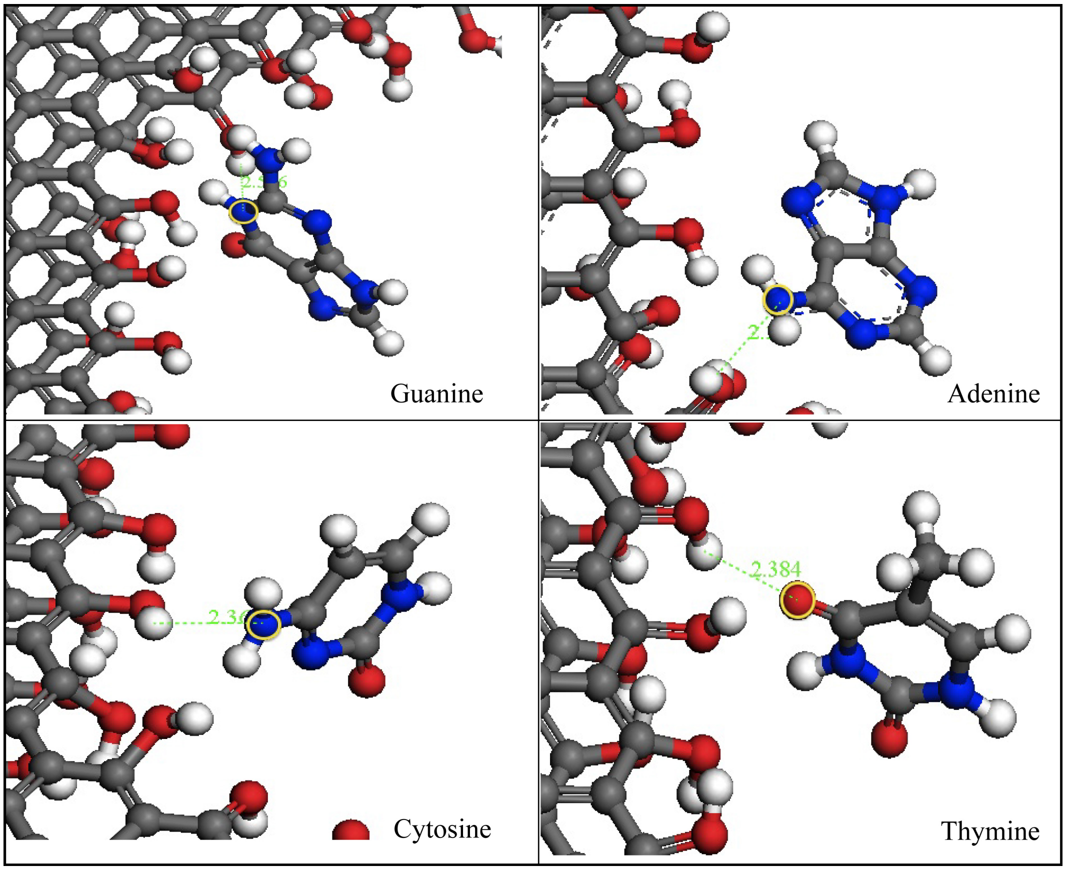

Figure S1: The distance measurement between the reference atom marked with a yellow circle and all other atoms of bilayer graphene.The shortest distances of each snapshot for four nucleobases taken from the MD simulation were saved.

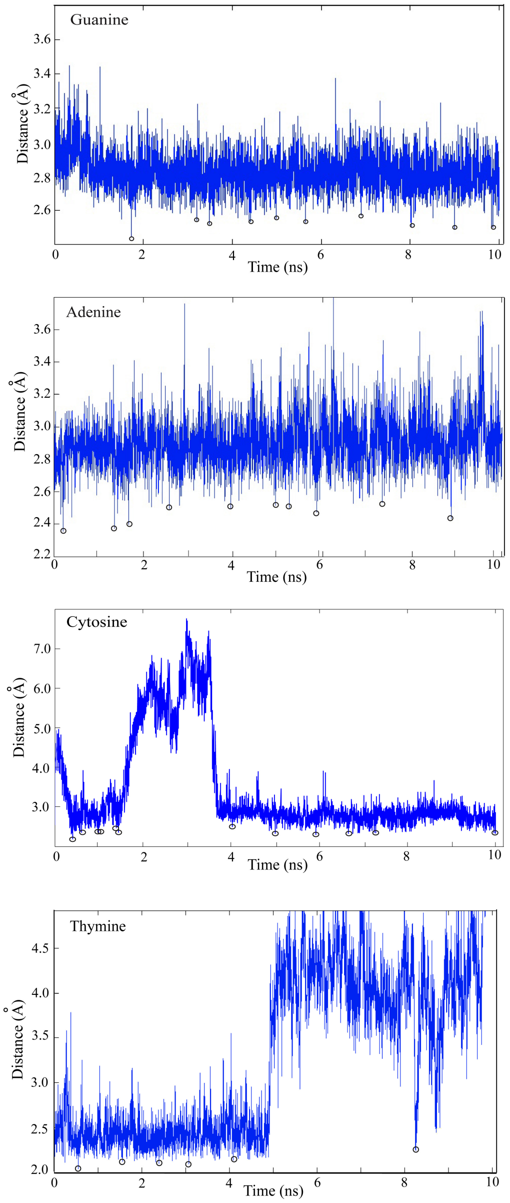

Figure S2: The shortest distance of each snapshot measured for simulation lengths of 10 ns. Some snapshots was selected for subsequent transport calculations, as marked with a black circle.

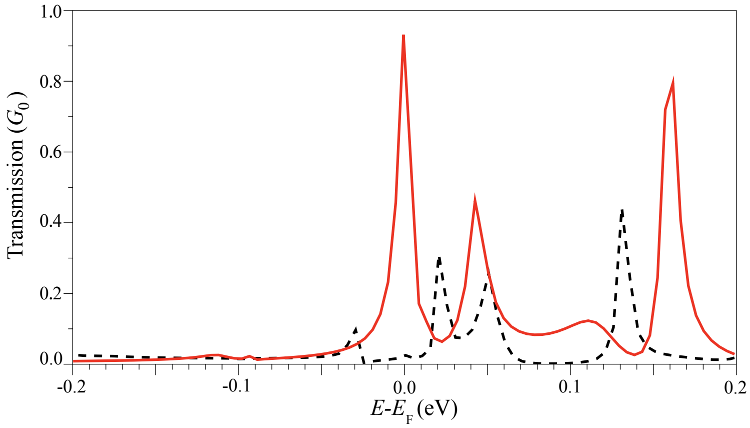

Figure S3: The transmission coefficients of the cytosine lying close (solid line) and far away (dashed line) from the pore edge.

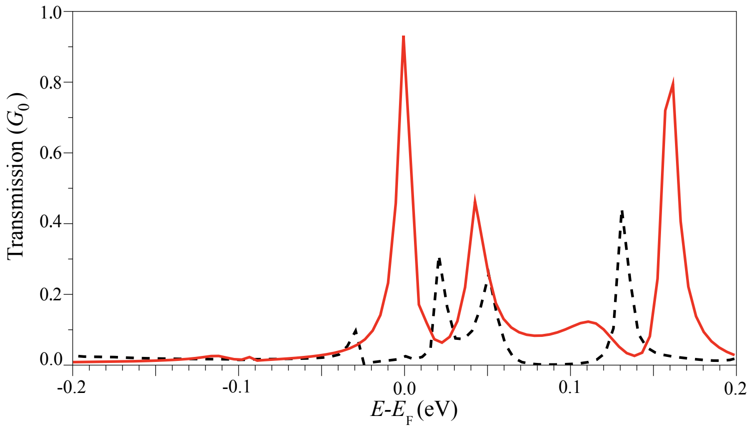

Figure S4: The transmission coefficients of the cytosine lying close (solid line) and far away (dashed line) from the pore edge.

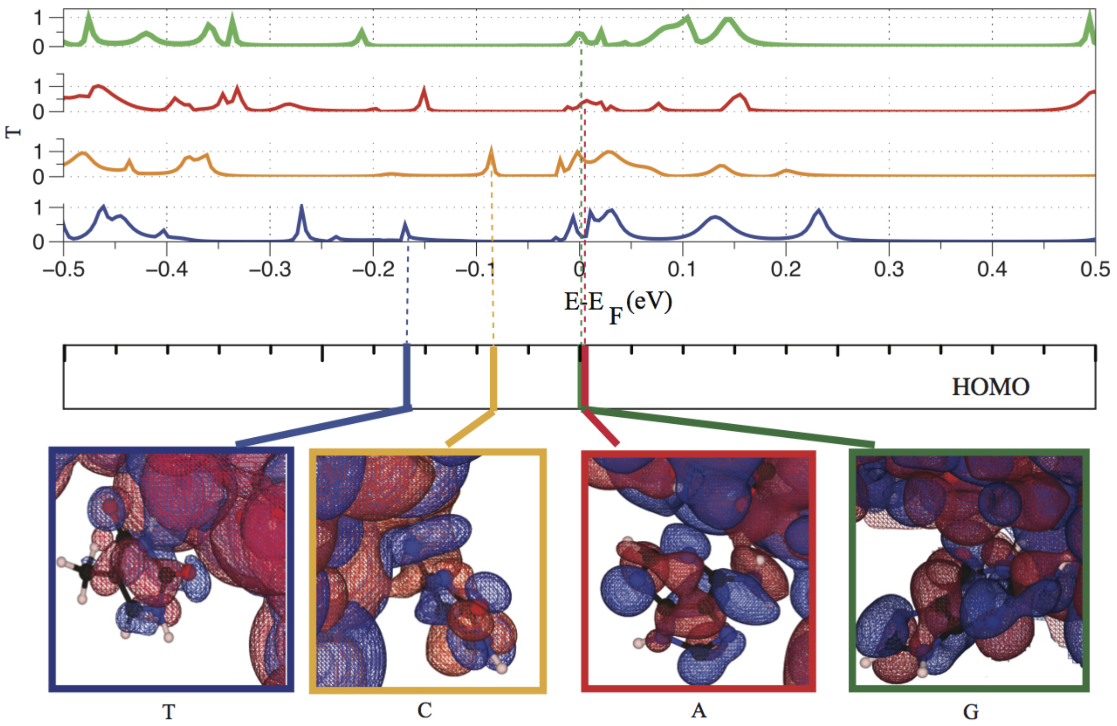

Figure S5: The transmission coefficients of the four target nucleobases (G, A, C, T) yielding maximum conductance. The resonance peaks associated with the HOMO of isolated nucleobases are identified.
